# Supplementary figures and images for: Multielectrode array characterization of human induced pluripotent stem cell derived neurons in co-culture with primary human astrocytes
Source: PLoS One. 2024 Jun 25;19(6):e0303901. doi: 10.1371/journal.pone.0303901 (PMC11198861; doi:10.1371/journal.pone.0303901)

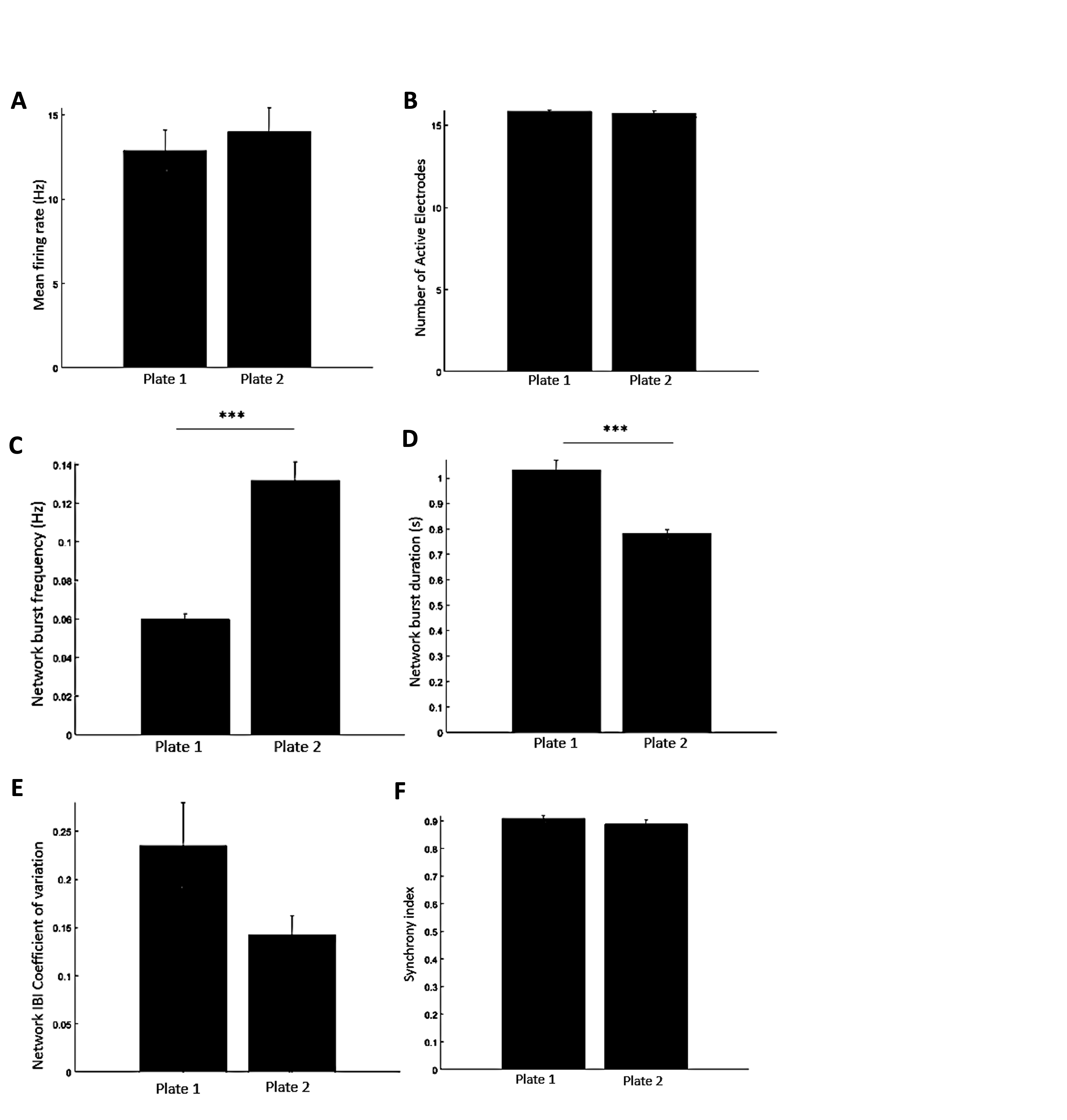

Supplement: S1 Fig — Characterized by (A) Mean firing rate (Hz) (B) Number of active electrodes (C) Network burst frequency (Hz) (D) Average network burst duration (s) (E) Network interburst interval coefficient of variation (F) Synchrony index. Statistical analysis was performed with an independent t-test. Error bar: SEM. N = 24 independent wells per plate. p-value < 0.05 *, p-value < 0.01 **, p-value < 0.001 ***. (TIFF) [file pone.0303901.s001.tiff]

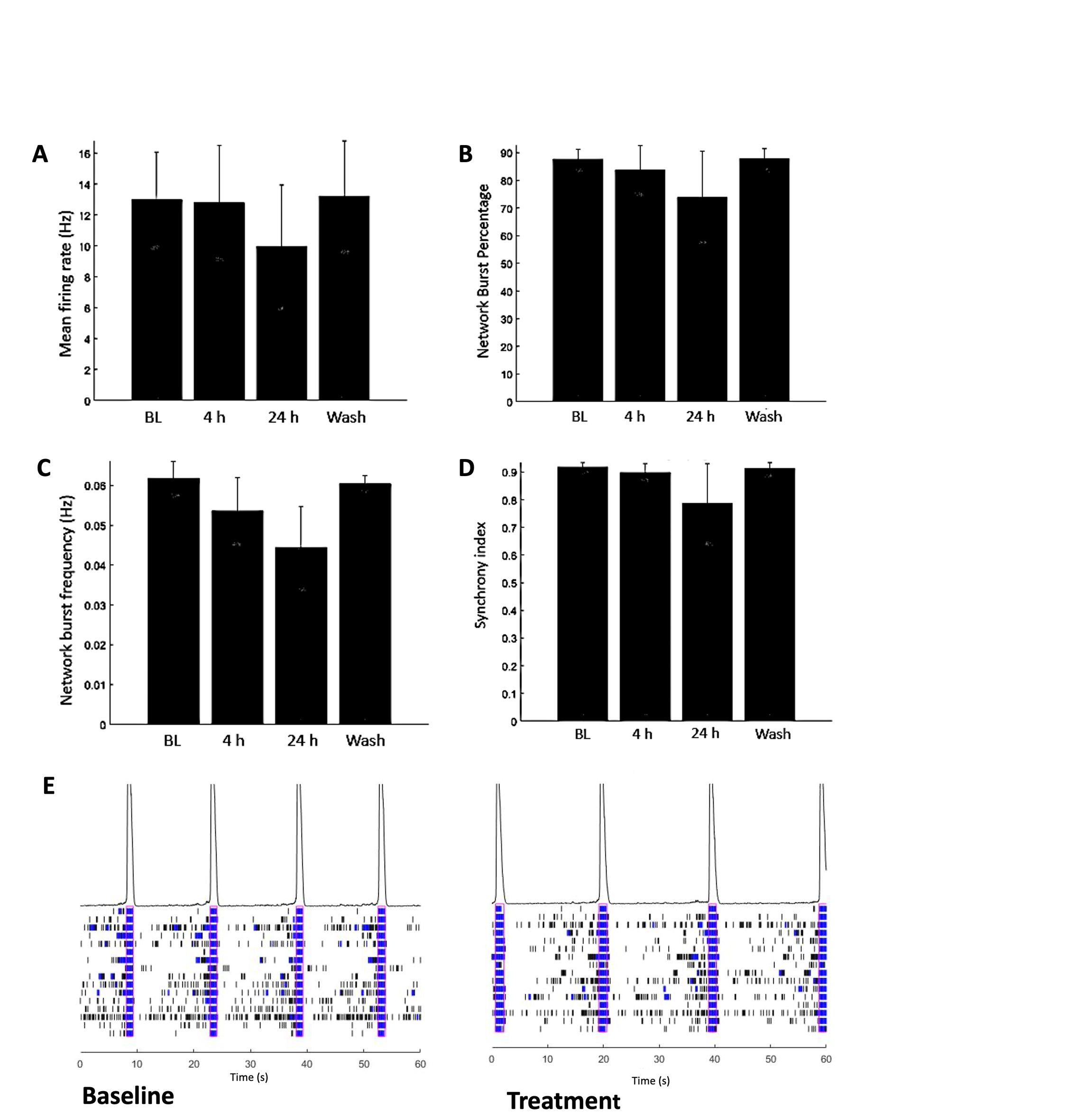

Supplement: S2 Fig — Quantifications of (A) Mean firing rate (Hz) (B) Network burst percentage (C) Network burst frequency (D) Synchrony index for baseline (BL), 4 hours and 24 hours after treatment, and after washout. (E) Representative raster plots and histograms of relative population firing rates for baseline and 24 hours after treatment Statistical analysis was performed with repeated measures ANOVA and Bonferroni corrections. Error bar: SEM. N = 4 independent wells. p-value < 0.05 *, p-value < 0.01 **, p-value < 0.001 ***. (TIF) [file pone.0303901.s002.tif]
